# Depression and Decay - A Case of Major Depressive Disorder with Psychotic Features in an Elderly Patient with Cancer

**DOI:** 10.1192/j.eurpsy.2023.1975

**Published:** 2023-07-19

**Authors:** E. Wee Yun Qing

**Affiliations:** Ministry of Health Holdings, Singapore

## Abstract

**Introduction:**

Major Depressive Disorder is a common psychiatric diagnosis among elderly patients with cancer. More than 10% of cancer patients suffer from Depression, which negatively impacts their quality of life. Mood symptoms are treated pharmacologically with antidepressant medications, while psychotic symptoms are treated with antipsychotic medications. Electroconvulsive Therapy may be initiated early for psychotic depression.

**Objectives:**

The objective of this case is to present a patient with Major Depressive Disorder, who developed psychotic symptoms after a diagnosis of malignancy, and to suggest early initiation of Electroconvulsive Therapy for treatment.

**Methods: Case Report:**

Ms G is a 68-year-old female who presented with low mood, worries, poor sleep, anhedonia and suicidal ideation. She complained of abdominal bloatedness and was reviewed by Gynaecology, who discovered a large ovarian mass, likely malignant in nature. Within days of the diagnosis, the patient developed nihilistic delusions, believing that her body was decaying and filled with poison. She was distressed and worried that the poison would spread to others around her. Risperidone was initiated and uptitrated, with limited improvement. As early surgical removal of the ovarian tumour was advised, it was essential that the patient’s mental state be quickly stabilised. She was hence initiated on Electroconvulsive Therapy (ECT). The patient underwent a total of 11 sessions of ECT. She was initially started on daily sessions, and the frequency gradually tapered.

**Results:**

The patient’s mental state improved. She was then continued on oral medications, and discharged home well to proceed with her Gynaecological surgery.

**Conclusions:**

This is a case of Major Depressive Disorder with psychotic symptoms that emerged after a diagnosis of malignancy. This case illustrates the importance of routine screening for psychotic symptoms, including nihilistic delusions, in elderly patients with Major Depressive Disorder who are newly diagnosed with cancer. It may be beneficial to consider early Electroconvulsive Therapy when treating such patients.

**Disclosure of Interest:**

None Declared

